# Childhood Bartter’s syndrome: An Indian case series

**DOI:** 10.4103/0971-4065.73455

**Published:** 2010-10

**Authors:** K. Sampathkumar, U. Muralidharan, A. Kannan, M. Ramakrishnan, R. Ajeshkumar

**Affiliations:** Department of Nephrology, Meenakshi Mission Hospital and Research Centre, Madurai - 625 107, India; 1 Department of Pediatrics, Meenakshi Mission Hospital and Research Centre, Madurai - 625 107, India

**Keywords:** Bartter’s syndrome, childhood, indomethacin, metabolic alkalosis

## Abstract

This is a retrospective analysis of children diagnosed with Bartter’s syndrome (BS) between 2001 and 2009 in our hospital. Seven children (six males) were diagnosed with BS. The mean age at presentation was 6.5 ± 4.9 months. The presenting features were failure to thrive,vomiting, polyuria, and dehydration. All children were normotensive at admission. The children exhibited alkalemia (pH, 7.58 ± 0.03), hypokalemia (serum potassium, 2.62 ± 0.47 mEq/l), hypochloremia (serum chloride, 82.83 ± 16.7 mEq/l), and hyponatremia (serum sodium, 126.85 ± 3.56 mEq/l). Disproportionate urinary wasting of sodium, potassium, and chloride were seen. The diagnosis was confirmed by elevated serum levels of both renin and aldosterone with normotension. Indomethacin or ibuprofen therapy resulted in marked improvement in general condition of these children. In conclusion, a high index of suspicion should be entertained in children with failure to thrive to diagnose BS. Therapy with NSAIDs leads to marked improvement in the general well being.

## Introduction

In 1962, Bartter *et al*. described a new disease entity in two African Americans who presented with metabolic alkalosis, hyperplasia of juxtaglomerular apparatus, and normotensive hyperaldosteronism.[1] Over the years, several phenotypic and genotypic variants of the original descriptions of Bartter’s syndrome (BS) have been identified. It is an uncommon inherited renal tubular disorder with hyponatremia, hypokalemia, hypochloremic metabolic alkalosis, hyperreninemia with normal blood pressure associated with increased urinary loss of sodium, potassium, calcium, and chloride. The primary defect in BS is an impairment in one of the transporters involved in sodium chloride reabsorption in the thick ascending limb of loop of Henle viz., Na-K-2Cl cotransporter (NKCC2) or apical K channel (ROMK) or basolateral chloride channel (ClCNKB).[2] BS is transmitted as an autosomal recessive disorder. The estimated prevalence is approximately 1 per million for BS in the western population. However, the prevalence of heterozygotes may be as high as 1 percent.[3] A history of consanguineous marriage is present in many families. Most cases of BS are present in neonates. Prenatally, neonatal BS can be diagnosed by finding elevated amniotic fluid chloride and aldosterone levels. Only isolated case reports but no case series have been reported so far from India.[4–8] This is the largest series of BS reported so far from India.

## Methods

A retrospective analysis of children diagnosed with BS was carried out from the departments of Nephrology and Pediatrics of Meenakshi Mission Hospital and Research Centre, Madurai, India during an 8-year period of 2001 to 2009. Based on the clinical, biochemical features and ultrasonographic findings, seven children were diagnosed of having BS. Their demographic, clinical, biochemical, and hormonal profiles were analyzed. Follow-up data and outcomes were recorded.

## Result

The clinical profiles of these children are described in Table 1.

There is a male predominance in our series with a male : female ratio of 6 : 1. The mean age at diagnosis was 6.5 ± 4.9 months. The predominant presentations were failure to thrive, vomiting, polyuria, and dehydration.

**Table 1:** Clinical profile of Bartter’s syndrome

Sex	Age at presentation (months)	Blood pressure (mmHg)	Presenting clinical features
M	3	96/60	Failure to thrive, dehydration
M	2	90/60	Vomiting, respiratory distress, convulsions
M	5	100/60	Failure to thrive, polyuria, dehydration
M	5	100/60	Failure to thrive, vomiting, fever, irritability, dehydration
M	12	90/60	Failure to thrive, respiratory distress
M	4	100/60	Failure to thrive, vomiting, dehydration
F	15	96/60	Recurrent vomiting, polyuria, failure to thrive, maternal polyhydramnios

This nonspecific symptom complex is common to many tropical diseases such as acute gastroenteritis, neonatal sepsis, meningoencephalitis, etc. Hence, the diagnosis can easily be missed if a high index of suspicion is not entertained and serum and urinary electrolytes are ordered and interpreted correctly.

Table 2describes the serum and urinary biochemical profile of BS.

**Table 2:** Blood and urinary biochemistry of Bartter’s syndrome

No	Blood pH	Serum sodium (mEq/l)	Serum potassium (mEq/l)	Serum bicarbonate (mEq/l)	Urine sodium (mEq/l)	Urine potassium (mEq/l)	Urine chloride (mEq/l)	Urine Ca/Cr (mg/mg)
1	7.56	120	1.8	49.5	154	31.9	155	0.3
2	7.53	127	2.5	45.7	110	12	NA	0.06
3	7.73	110	1.5	44.6	-	-	97	0.45
4	7.61	131	1.7	53.7	113	9	120	NA
5	7.56	136	2.9	30.1	88	49	90	0.28
6	7.48	137	5.2	26.5	7	7.5	110	0.43
7	7.65	127	2.8	26.5	44	20	45	NA
Mean	7.58 ± 0.03	126.85 ± 3.56	2.62 ± 0.47	39.51 ± 4.3	86 ± 21.5	21.56 ± 6.6	102.83 ± 14.8	0.30

All children were alkalemic at presentation. Both serum sodium levels and serum chloride levels were low. The urinary electrolyte profile showed sodium, chloride, and potassium wasting which is characteristic of BS.

The normal reference values of serum renin and aldosterone were 0.15 to 3.95 ng/ml/h and 10 to 160 ng/l, respectively. These children showed elevated serum levels of both renin and aldosterone, as shown in Table 3.

**Table 3:** Serum renin, aldosterone, and renal ultrasonogram findings

S.No	Serum renin (ng/ml/hr)	Serum aldosterone (ng/l)	Ultrasound abdomen finding
1	8.5	330	Normal
2	187	848.7	Bilateral medical renal disease
3	6.05	1400	Normal
4	3.23	86.3	Bilateral mildly increased renal cortical echoes
5	8.6	752	Normal
6	40.71	967	Nephrocalcinosis
7	4	135	Normal
Mean	36.8 ± 42.3	645 ± 482.7	

Ultrasound examination of abdomen showed nephrocalcinosis in one child whose urine calcium excretion was elevated (calcium/creatinine ratio was 0.43), bilateral mildly increased renal internal echoes in three children, and normal study in the remaining four.

**Figure 1:**
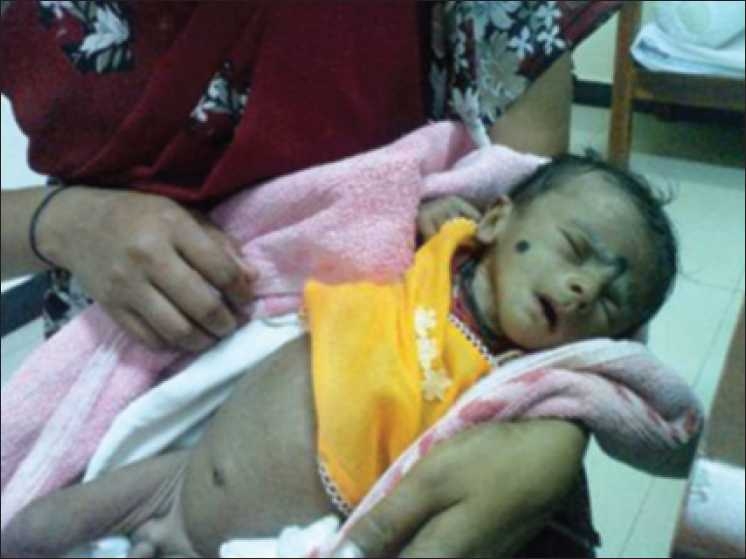
Bartter’s child before treatment showing severe dehydration

**Figure 2:**
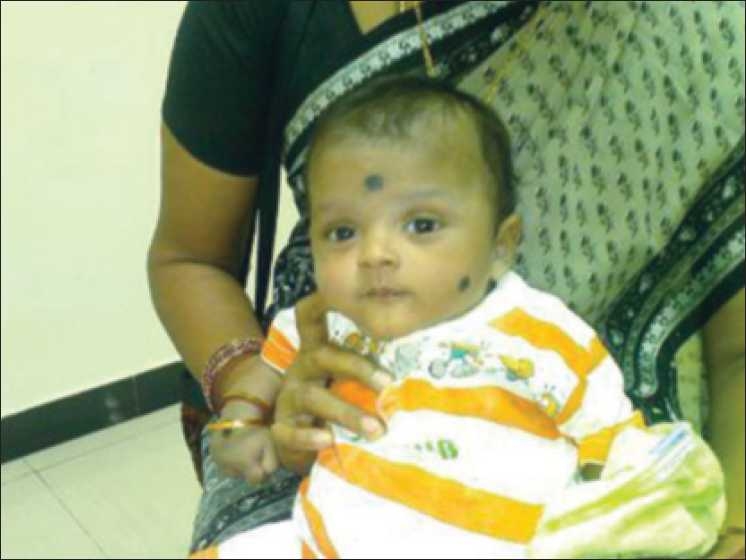
Same child four months after treatment

These children initially were treated with isotonic saline and potassium chloride replacement. After 48 hours of parenteral therapy, these babies were started on oral potassium chloride supplements. Three babies were treated with oral Ibuprofen therapy in a dose of 15 mg/kg/day. Three were given indomethacin therapy, in a dose of 2 mg/kg/day. Four babies were on regular follow-up over a period of 3 to 12 months. Weight gain was sufficient to achieve catch-up growth in two babies, whereas the rest were lagging behind in growth. Figures 1 and 2 captures the marked improvement in the general condition of a child after starting therapy. Two babies died soon after diagnosis due to aspiration pneumonia before specific therapy could be started. One baby was lost to follow-up.

## Discussion

To our knowledge, ours is the first case series to document the clinical profile of BS from India. The exact incidence of BS is not known. In Costa Rica, incidence of neonatal Bartter’s from live births is estimated as one per 1.2 million.[9] In Kuwait, it was estimated as 1.7 per million population and in Sweden as 1.2 per million population.[10] The age at admission ranged from 2 to 15 months, with the mean age of 6.45 months which compares with the two published case series.[5,7] In our study, most of the children were male, whereas Abdel-al *et al*.’s series had female predominance and Dillon *et al*.’s series showed equal sex distribution.[5,7]

In our study, history of consanguinity was present in two babies, polyhydramnios and prematurity were present in two. In the study by Abdel-al *et al*, both consanguinity and familial history among the patients were high (69 and 54%, respectively).[7] Shalev *et al*. from Israel studied the neonatal variant of BS with deafness. They analyzed retrospectively the data from 13 infants with BS and nerve deafness who were born during a 20-year period. They found that all pregnancies were complicated by polyhydramnios and premature birth.[9] The commonest symptoms noticed in our study were failure to thrive, vomiting, and dehydration. Six children had failure to thrive, four had vomiting, and another four had dehydration at admission. Other presenting features were fever, irritability, respiratory difficulty, and convulsion.

The process of active sodium chloride transport in the thick ascending limb of loop of Henle segment is mediated at the luminal membrane by the loop diuretic-sensitive Na-K-2Cl cotransporter that results in sodium chloride entry into the tubular cells and by potassium channels that permit reabsorbed potassium to leak back into the lumen for continued Na-K-2Cl cotransport. At the basolateral membrane, chloride channels permit the chloride that has entered the cell to exit and be returned to the systemic circulation. BS can result from a defect in the gene for any one of these transporters, illustrating the requirement for their integrated function in loop transport. Defects in the Na-K-2Cl cotransporter, the luminal potassium channel, and the basolateral chloride channel are known as BS types I, II, and III, respectively. Recent advance in molecular diagnostics have revealed that BS results from mutations in five distinct genes that alter the function of ion channels and transporters in the distal nephron. Diminished chloride and sodium reabsorption leads to low serum sodium, which in turn leads to stimulation of renin-angiotensin-aldosterone axis with resultant urinary potassium and hydrogen loss. Low serum potassium in turn stimulates release of prostaglandins (PGE2 and PGI2). There is elevation of plasma bradykinin and renal kallikrein. The hypertensive effect of angiotensin II and aldosterone is neutralized by the vasodepressor effect of PGE2 and bradykinin, thus explaining the normal blood pressure.

We had one case with elevated creatinine value of 2.6 mg/dl, which normalized on the next day itself. One of the baby’s ultrasound showed nephrocalcinosis. In Abdel-al *et al*.’s study, 11patients (85%) had growth failure, two had nephrocalcinosis (15%), and one had renal failure.[10] The study by Garel *et al*. showed nephrocalcinosis in all the five children by computed tomography scan and ultrasonography.[11]

The study by Abdel-al *et al* also showed hypokalemia, hypochloremia, metabolic alkalosis, and hyperreninemia in all the patients.[10] None of the children in our series were hypertensive despite high renin and aldosterone levels.

Renal biopsy was not performed in our children. The study by Shalev *et al*. revealed mild focal tubulointerstitial fibrosis and minimal mesangial proliferation but no glomerulosclerosis in kidney biopsies from two 7-year-old patients.[12] We had one case with the complication of nephrocalcinosis, and one child had renal failure which resolved after fluid therapy.

Dillon *et al*. used indomethacin in six of ten children for 6 to 24 months.[9] In the study by Abdel-al *et al*., all patients were treated with an aldosterone antagonist (spironolactone) and a prostaglandin synthetase inhibitor (indomethacin or aspirin) sequentially.[10] Growth hormone therapy was not given to our children. But studies have showed that nearly all patients with BS have growth retardation and are given growth hormone therapy along with potassium and indomethacin. A case report showed an association between BS and isolated familial growth hormone deficiency, with growth hormone therapy providing good results.[13]

Abdel *et al*. showed significant catch-up growth in 30.76% and increase in serum potassium value in 61.53%. One baby died (7.69%) of severe pneumonia with respiratory failure from hypokalemic myopathy.[10] The study by Dillon *et al*. showed catch-up growth in all patients treated with indomethacin therapy with remarkable clinical and biochemical improvement.[9] Usually prognosis in many cases is good, with patients being able to lead fairly normal lives.[6]

Genetic studies were not done in any of our children due to nonavailability of such specialized laboratories in our region. There is no direct correlation between the clinical phenotype and the underlying genotypic abnormality, even with well-characterized defects in a single transporter. However, more severe and earlier clinical manifestations may be seen with mutations leading to defects in Na-K-2Cl cotransporter and the luminal potassium channel.[14]

## Conclusion

BS should be suspected in any child with history of failure to thrive and metabolic alkalosis. Early diagnosis and treatment with NSAIDs are lifesaving.
